# Incidental Finding of an Infarcted Epiploic Appendage Attached to the Sigmoid Colon

**DOI:** 10.31486/toj.24.0123

**Published:** 2025

**Authors:** Ifeanyi K. Uche, Alec A. Hirsch

**Affiliations:** ^1^ Louisiana State University Health Sciences Center School of Medicine, New Orleans, LA; ^2^ Woman's Surgical Specialty Group, Baton Rouge, LA

**Keywords:** *Abdominal pain*, *colon–sigmoid*, *epiploic appendagitis*

## Abstract

**Background:**

Epiploic appendagitis is a condition usually caused by infarction of the epiploic appendages, small outpouchings of adipose tissue found on the serosal surface of the colon. Epiploic appendagitis is a rare cause of acute lower abdominal pain, but the condition does not have any specific presenting clinical features and is often misdiagnosed as acute diverticulitis, appendicitis, or other gastrointestinal disorder.

**Case Report:**

A 53-year-old female presented with abnormal uterine bleeding and pelvic pain and was seeking definitive surgical management. During the patient's total laparoscopic hysterectomy with bilateral salpingo-oophorectomy, an infarcted epiploic appendage attached to the sigmoid colon was found. An intraoperative general surgery consultation was obtained, and the infarcted epiploic appendage was laparoscopically excised.

**Conclusion:**

This case provides information about epiploic appendagitis, a rare cause of abdominal pain that can clinically mimic other acute or subacute disorders. The goal is to increase awareness of this rare intra-abdominal condition.

## INTRODUCTION

Epiploic appendages are small adipose outpouchings typically located on the outer surface of the large intestine.^1^ Because of their pedunculated morphology, limited blood supply, and excessive mobility, these appendages are susceptible to torsion and ischemic infarction that can result in a condition known as epiploic appendagitis.^2,3^ While epiploic appendagitis can occur in any segment of the colon, it is most frequently observed in the sigmoid colon.^3^ The clinical presentation includes acute abdominal pain localized to either the left or right lower quadrant, and the condition is often misdiagnosed as acute diverticulitis, appendicitis, or other gastrointestinal disorder.^4^

Because of the lack of specific clinical features, epiploic appendagitis is typically diagnosed with abdominal computed tomography (CT) scans.^3^ However, ultrasound and magnetic resonance imaging (MRI) have also been used to diagnose epiploic appendagitis.^5-8^

We report a rare incidental finding of a torsed epiploic appendage arising from the antimesenteric border of the sigmoid colon in a patient who presented with abnormal uterine bleeding and pelvic pain.

## CASE REPORT

A 53-year-old African American female with a body mass index of 31.64 kg/m^2^ and a medical history of anemia, type 2 diabetes, hypertension, hyperlipidemia, menorrhagia, and pelvic pain presented with abnormal uterine bleeding and pelvic pain. Her surgical history included salpingectomy for ectopic pregnancy, tubal ligation for contraception, and reduction mammaplasty. The patient reported that her menstrual cycle occurs every month, lasts 7 days, and involves heavy bleeding that saturates 4 pads per day. Throughout the month, the patient reported also experiencing back pain and pelvic pressure. Her medical record showed multiple emergency department visits during which she reported pelvic pain, principally localized to the right lower quadrant. The pain was not associated with nausea, vomiting, fever, urinary symptoms, or change in bowel habits.

The patient was scheduled for a total laparoscopic hysterectomy with bilateral salpingo-oophorectomy. The procedure was performed under general endotracheal anesthesia with the patient in dorsal lithotomy position. For the procedure, multiple 5-mm cannulas were inserted into the peritoneal cavity, and a small, inflamed, multilobulated, reddish-pink mass was noted near the sigmoid colon (Figure). Intraoperative general surgery consultation was obtained, as the lesion appeared to be a torsed epiploic appendage arising from the antimesenteric border of the sigmoid colon.

**Figure.**
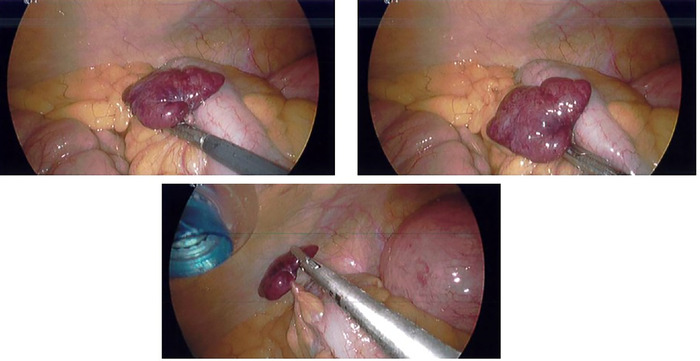
Laparoscopic views of the infarcted epiploic appendage on the sigmoid colon before excision.

The epiploic appendage was placed under gentle traction using an atraumatic grasping instrument. The vascular pedicle was twisted. A clip was applied to the vascular pedicle just above the sigmoid colon, and the pedicle was transected between the clip and the lesion. The lesion was placed in an Endo Catch specimen retrieval pouch (Medtronic), removed through the right lower quadrant port site, and sent to Pathology. No blood loss occurred during the procedure. The gynecology team then proceeded with the total laparoscopic hysterectomy with bilateral salpingo-oophorectomy.

Pathology identified the excised infarcted epiploic appendage as a 3.8 × 3.0 × 0.8-cm section of dusky, congested fatty tissue.

The patient tolerated the procedures well and was discharged home the same day. Her postoperative recovery was uneventful and quick; she reported only mild pain at the incision sites and nausea. She had no vomiting or fever, and her bowel and bladder functions remained normal. Her pain was well controlled with nonsteroidal anti-inflammatory drugs (NSAIDs).

At her 6-week postoperative follow-up, the patient reported complete resolution of pelvic pain and abnormal uterine bleeding. She denied any fever, vaginal bleeding, discharge, or bowel or urinary complaints at the postoperative follow-up.

## DISCUSSION

As noted previously, epiploic appendages can be distributed throughout the colon but are more often concentrated in the sigmoid colon.^3^ They are absent from the rectum.^3,5^ Epiploic appendages usually measure 1 to 2 cm in length and 2 to 5 cm in thickness.^3^ They receive blood supply from 1 or 2 small end arteries branching from the vasa recta longa of the colon and are drained by a single vein.^9,10^ Epiploic appendagitis occurs when an epiploic appendage becomes torsed, causing obstruction of the vascular or venous supply that leads to ischemia.^5,11^ Epiploic appendagitis can also occur from embolic or thrombotic causes.^1^ Epiploic appendagitis is a rare condition, with a frequency of 1.3% and an incidence of 8.8 cases per million annually.^12^ However, the incidence may be increasing because of advancements in diagnostic imaging.

Epiploic appendagitis is associated with obesity, hernia, and strenuous exercise^5,8,11^ and commonly occurs in people in the fourth and fifth decades of life, predominantly in males.^8^ Epiploic appendagitis typically presents with sudden onset of localized left or right lower quadrant acute abdominal pain. Most patients describe the pain as a dull, nonmigratory, localized, constant ache.^13^ Epiploic appendagitis is rarely associated with other symptoms such as nausea, vomiting, diarrhea, constipation, and fever.^11^ Therefore, epiploic appendagitis should be considered as a potential diagnosis in patients with acute localized nonmigratory abdominal pain in the absence of the aforementioned symptoms.

Patients with epiploic appendagitis typically have normal vital signs.^11^ Laboratory workup is generally unremarkable with a slight increase in serum levels of C-reactive protein and leukocyte count.^10,14^ Diagnosis can be made via CT scan, ultrasound, and MRI.^5-8^ The classic CT findings of epiploic appendagitis are (1) an ovoid lesion with fat attenuation surrounded by a thin hyperdense rim (hyperattenuating ring sign), (2) mild bowel wall thickening, and (3) central high-attenuation focus within the fatty lesion (central dot sign), indicative of a thrombosed vein in the inflamed epiploic appendage.^1^ On ultrasound, an epiploic appendagitis lesion appears as a noncompressible, solid, hyperechoic mass attached to the colon.^15^ No blood flow to the lesion is observed on color Doppler signaling.^15^ MRI in patients with epiploic appendagitis shows an oval-shaped fat mass with a central dot on both T1- and T2-weighted images, with an enhancing rim seen on post-gadolinium T1-weighted fat-saturated images.^16^

The clinical features of epiploic appendagitis often resemble those of diverticulitis or acute appendicitis.^5,13^ Other conditions that may mimic epiploic appendagitis include acute omental infarction, mesenteric panniculitis, sclerosing mesenteritis, and primary tumors or metastases involving the mesocolon.^5^ On CT, acute diverticulitis, in contrast to epiploic appendagitis, shows colonic diverticula with associated inflammation or abscess formation in the mesocolon and colonic wall thickening.^5^ Furthermore, patients with acute diverticulitis are more likely to present with symptoms such as fever, nausea, vomiting, elevated white blood cell count, and rebound tenderness that are uncommon in patients with epiploic appendagitis.^5^ On CT, an omental infarction usually shows as a larger lesion compared to epiploic appendagitis and lacks the hyperattenuating ring sign seen in epiploic appendagitis. Additionally, omental infarction primarily affects pediatric patients, whereas epiploic appendagitis is more commonly seen in adults.^5^ Sclerosing mesenteritis typically occurs in individuals in the sixth to seventh decades of life and has a higher incidence in males. Sclerosing mesenteritis can present with symptoms such as abdominal pain, fever, nausea, vomiting, diarrhea, and weight loss.^5^ Nevertheless, taking a thorough history and performing a physical examination, including a gynecologic examination for females, are essential to determine the cause of abdominal pain. For females, therefore, the differential diagnosis should include conditions such as ovarian torsion, ovarian cyst rupture, and ectopic pregnancy. Our patient's pain was possibly caused by epiploic appendagitis but may have been exacerbated by her abnormal uterine bleeding disorder.

Epiploic appendagitis is usually self-limiting and can be treated conservatively with NSAIDs.^5,7^ Patients with epiploic appendagitis have also been treated with a combination of NSAIDs and antibiotics,^7^ although the treatment will depend on the patient's symptoms. Symptoms typically resolve within several days to weeks of conservative management. If symptoms do not improve, surgery is warranted.^7,8^ Surgical excision of inflamed epiploic appendages is the most effective treatment for patients with recurrent epiploic appendagitis and for patients with complications such as intussusception, adhesions, and abscess formation.^8^

## CONCLUSION

In our case, epiploic appendagitis was an incidental finding in a patient who presented with abnormal uterine bleeding and pelvic pain. This case study is aimed at providing information about epiploic appendagitis, which is often mistaken as another more severe cause of abdominal pain. Our goal is to increase awareness of this rare intra-abdominal condition.

## Notes

*This article meets the Accreditation Council for Graduate Medical Education and the American Board of Medical Specialties Maintenance of Certification competencies for Patient Care and Medical Knowledge.*
